# *LEADR*, a p63 target, dampens interferon signalling in bladder cancer

**DOI:** 10.1038/s41420-025-02546-1

**Published:** 2025-06-03

**Authors:** Damiano Barnaba, Mariacristina Franzese Canonico, Manuela Helmer-Citterich, Paolo Gandellini, Gerry Melino, Artem Smirnov, Eleonora Candi

**Affiliations:** 1 Department of Experimental Medicine, University of Rome Tor Vergata, Rome, Italy; 2 Biology Department, University of Rome Tor Vergata, Rome, Italy; 3 Department of Biosciences, University of Milan, Milan, Italy; 4 Istituto Dermopatico Immacolata (IDI-IRCCS), Rome, Italy

**Keywords:** Long non-coding RNAs, Transcriptional regulatory elements

## Abstract

Bladder cancer affects over half a million people worldwide each year. Recent advances in early detection allowed a successful management of non-aggressive cancers, yet the recurrence rate remains high. Aggressive muscle-invasive bladder tumours are life-threatening and challenging to cure. Therefore, understanding of key molecular pathways involved in cancer progression is critical for developing of new personalised targeted therapies. Recently, non-coding RNAs (ncRNAs) have emerged as key regulators orchestrating complex biological processes in cancer, yet their function is not fully understood. Here, we compare non-muscle invasive and muscle invasive cell lines and identify a ncRNA gene *MIR205HG* and its transcript *LEADR* among the top ncRNAs downregulated in muscle invasive urothelial tumours. We show that *LEADR* expression is epigenetically regulated by master transcription factor p63. *LEADR* is localised in the nuclei of non-muscle invasive bladder cancer cells where it dampens hyperactivation of interferon stimulated genes possibly increasing sensitivity of bladder cancer cells to interferon signalling. These findings uncover an anti-tumoral role of non-coding RNA *LEADR* in mediating immune response in bladder cancer.

## Introduction

Based on the latest GLOBOCAN data [1], bladder cancer (BLCA) is the ninth most common cancer worldwide, it ranks thirteenth in mortality rate and affects men more than women. The majority of bladder cancers originate from the urothelium, giving rise to urothelial carcinomas that can be either non-muscle invasive (NMIBC) or muscle invasive (MIBC) tumours. In approximately 75% of the patients diagnosed with bladder cancer, the tumour is confined to the bladder mucosa or to the lamina propria, while the remaining 25% of the tumours are invasive into the muscularis propria [2]. If diagnosed at early stages, transurethral resection (TUR or TURBT, transurethral resection of bladder tumour) allows a correct tumour staging and determines eventual adjuvant therapies, such as mitomycin C, gemcitabine or the immune adjuvant Bacillus Calmette–Guerin (BCG) [3, 4]. MIBC treatment includes radiotherapy [5, 6], neoadjuvant or adjuvant chemotherapy to lower the risk of recurrence, yet radical cystectomy with pelvic lymph node dissection (PLND) remains a standard surgical approach. Despite the available treatment options, the recurrence rate of MIBC is substantial, highlighting the need for deeper understanding the molecular basis underlying MIBC to improve patients survival.

In recent years, non-coding RNAs (ncRNAs) have emerged as key regulators of numerous biological processes, including cell differentiation, proliferation, and metabolism [7–13]. Over 120.000 ncRNAs have been annotated but the complex network of ncRNAs is still under intensive investigation. ncRNAs can be localised both in cytoplasm and nucleus and can regulate a variety of cellular pathways including chromatin remodelling and gene expression [14].

Alterations in expression of various ncRNAs can lead to cell transformation and tumorigenesis as well to cancer progression [15–19]. Several ncRNAs involved in bladder cancer progression have been identified, for instance urothelial carcinoma associated 1 (UCA1) [20] and ZEB2-AS1 [21] which are up-regulated in bladder tumours. Non-coding RNAs (ncRNAs) have been suggested as promising diagnostic biomarkers for the early detection of urothelial carcinoma and as valuable tools for assessing bladder cancer stage and prognosis [22]. Yet, it is not clear which ncRNAs are involved in the progression from NMIBC to MBIC cancers.

Here, we identify *MIR205HG* and its transcript *LEADR* as one of the most enriched ncRNAs in non-muscle invasive bladder cancer. The *MIR205HG* transcript gives rise to a micro-RNA *miR-205* and a Long Epithelial Alu-interacting Differentiation-related RNA (*LEADR*) lncRNA. While the role of miR-205 is well established [23], the function of *LEADR* remains poorly understood. Our findings demonstrate that in non-muscle invasive bladder cancer, LEADR is epigenetically regulated by p63 and functions as a negative regulator of the interferon pathway.

## Results

### *LEADR* is highly expressed in non-invasive bladder cancer

**Fig. 1:**
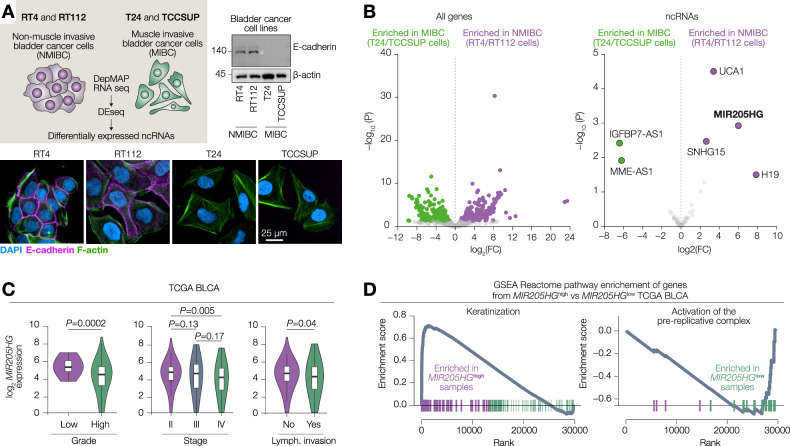
*LEADR* is highly expressed in non-invasive bladder cancer. **A** (Top) Schematic showing the in vitro model to identify differentially expressed non-coding RNAs in non-muscle invasive vs muscle invasive bladder cancer. (Right) Western blot of e-cadherin expression in the panel of BLCA cell lines. β-actin as loading control. (Bottom) Immunofluorescence analysis by confocal microscopy of e-cadherin and F-actin expression in the same cell lines. **B** Volcano plots showing differential expression of either all genes or ncRNAs only in NMIBC vs MIBC cell lines. Genes with abs(log_2_FC)>1 and *P* < 0.05 were considered significantly modulated. **C** *MIR205HG* log_2_ RNA expression in TCGA BLCA samples based on the grade, stage or lymphovascular invasion positivity. *P* by Kolmogorov-Smirnov (“grade” and “invasion”) or one-way ANOVA (“stage”) tests. **D** GSEA Reactome pathway enrichment of genes differentially expressed in *MIR205HG*-high vs *MIR205HG*-low TCGA BLCA tumours. The top up- and down-regulated pathways are shown.

To identify differentially expressed ncRNAs in low stage vs high stages bladder tumours, we chose two non-muscle invasive (RT4 and RT112) and two muscle-invasive (T24 and TCCSUP) bladder cancer cell lines. Non-muscle invasive cells in vitro resembled epithelial phenotype and expressed high levels of junctional e-cadherin while muscle-invasive cells had mesenchymal appearance and were negative for e-cadherin (Fig. 1A). We then interrogated total RNA seq of these cell lines from Cancer Cell Line Encyclopaedia. By applying DEseq2, we identified differentially expressed ncRNAs. Four ncRNAs, including *H19*, *UCA1*, *MIR205HG*, and *SNHG15* were strongly expressed in non-muscle invasive cells (Fig. 1B). Of note, *H19* [24], *UCA1* [25] and *SNHG15* [26] have been recently studied in bladder cancer. *MIR205HG* is a host gene for microRNA *miR-205*. Of note, *miR-205* has been demonstrated to inhibit EMT by repressing ZEB1 in bladder cancer [27]. Yet, the function of the host gene itself in bladder cancer remains unknown. Therefore, we decided to investigate the role of the *miR-205*-independent spliced transcript of *MIR205HG* hereinafter referred to as *LEADR*. By interrogating TCGA bladder cancer cohort, we observed that *MIR205HG* expression was decreased in high grade and high stage tumours, as well as in patients with lymphovascular invasion (Fig. 1C). We then divided TCGA BLCA patients based on *MIR205HG* expression and performed differential expression analysis. *MIR205HG*^high^ tumours were enriched for genes related to epithelial pathways including keratinization, ECM interactions and collagen formation. On the contrary, genes from *MIR205HG*^low^ tumours were associated with replication, unfolded protein response and nuclear receptor transcription (Fig. 1D and Supplementary Fig. S1A). Altogether, *MIR205HG/LEADR* gene is one of the top ncRNAs downregulated during bladder cancer progression.

### p63 regulates *LEADR* expression in bladder cancer

**Fig. 2:**
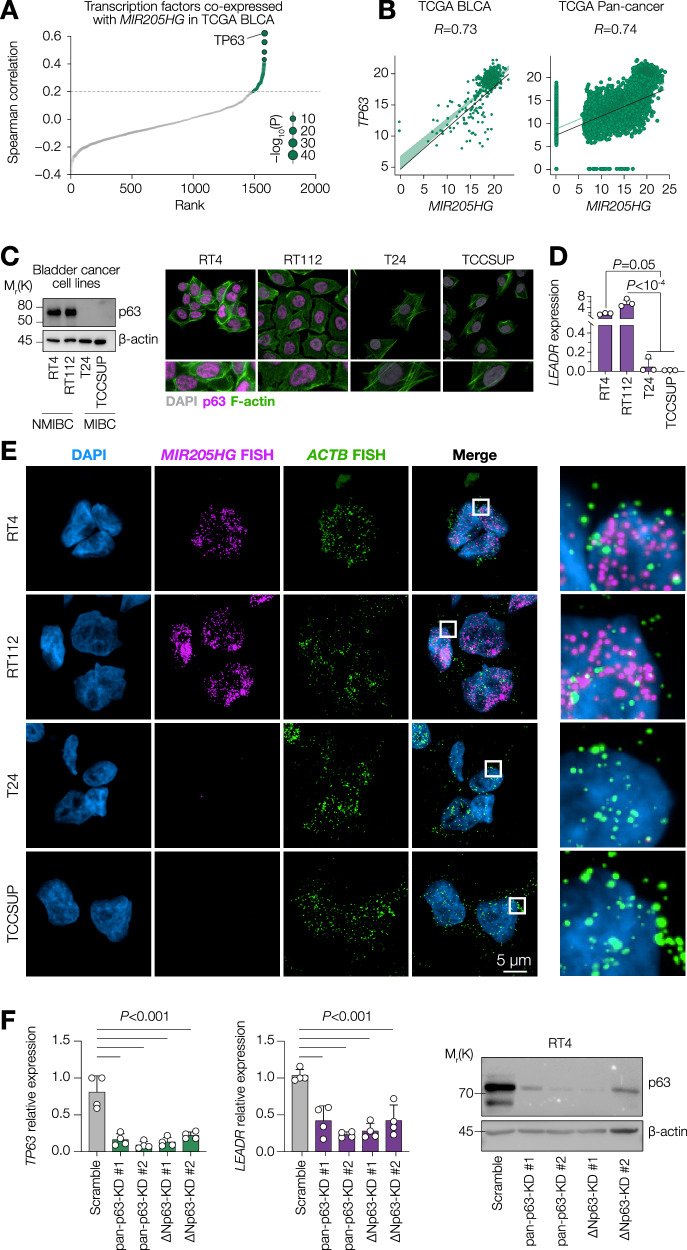
p63 regulates *LEADR* expression in bladder cancer. **A** Dot plot showing Spearman correlation between *MIR205HG* RNA expression and expression of known human transcription factors in TCGA BLCA samples. **B** Dot plot showing expression of *MIR205HG* and *TP63* in either TCGA BLCA of TCGA Pan-cancer samples. **C** (Left) Western blot of p63 levels in the panel of BLCA cell lines. β-actin as loading control. (Right) Immunofluorescence analysis by confocal microscopy of p63 and F-actin expression in the same cell lines. **D** RT-qPCR analysis of *LEADR* expression in the same cell lines. *n* = 3 (biological replicates). *P* by one-way ANOVA. **E** RNA FISH analysis by confocal microscopy of *LEADR* in the cell lines from (**D**). ACTB as a positive control. **F** RT-qPCR analysis of p63 and *LEADR* expression in RT4 cell knocked-down for p63 using distinct siRNAs. *n* = 4 (biological replicates). *P* by one-way ANOVA. Western blot on the right confirms an efficient knock-down of p63. β-actin as loading control.

To understand the transcriptional regulation of *LEADR*, we ranked all known human transcription factors based on their co-expression with *MIR205HG* in TCGA bladder cancer cohort and identified p63 as the top hit (Fig. 2A). Moreover, *MIR205HG* resulted to be the most co-expressed and the strongest potential ncRNA target of p63 based on the target score of TargetGeneReg algorithm [28] (Supplementary Fig. S2A). Indeed, *MIR205HG* expression was strongly correlated with *TP63* both in bladder cancer and across all TCGA cancer types (Fig. 2B and Supplementary Fig. S2B). These observations are in line with previous reports of p63 regulating miR-205 and *LEADR* in prostate cancer [29]. We then confirmed that p63 and *LEADR* transcript were co-expressed in our panel of bladder cancer cell lines by using *LEADR*-specific primers. Predominantly N-truncated 75 kDa ΔNp63 isoform was detected exclusively in the nuclei of non-muscle invasive RT4 and RT112 bladder cancer cell lines (Fig. 2C). Accordingly, high levels of *LEADR* were detected in RT4 and RT112, but not T24 or TCCSUP cell lines (Fig. 2D). Of note, *LEADR* was localised within nuclei of tumour cells as assessed by fluorescence in situ hybridization (FISH) using *MIR205HG* or control *ACTB* (β-actin) probes, whereas the nuclei of T24 and TCCSUP cells were negative for *MIR205HG* (Fig. 2E). To understand whether p63 regulated *LEADR* expression, we used a set of siRNAs either against all transcripts of *TP63* gene or specifically ΔΝp63 isoforms (Fig. 2F). All the siRNA led to a dramatic decrease of *LEADR* expression in RT4 cells. We further confirmed these data also in RT112 and SW780 non-muscle invasive cells using a pan-p63 siRNA (Supplementary Fig. S2C). Collectively, we show that p63 controls *LEADR* expression in non-muscle invasive bladder cancer.

### *LEADR* is epigenetically repressed in advanced bladder cancer

**Fig. 3:**
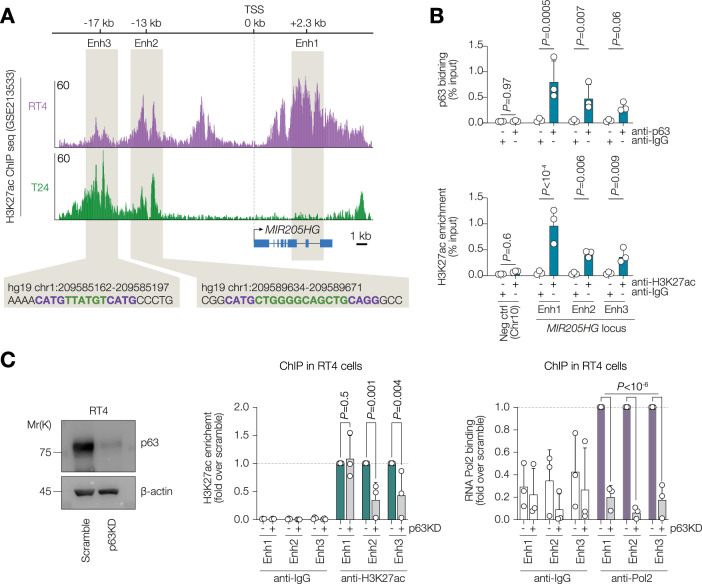
*LEADR* is epigenetically repressed in advanced bladder cancer. **A** UCSC genome browser screenshot of H3K27ac ChIP seq signal tracks in RT4 and T24 from the GSE213533 study. The *MIR205HG* locus is shown. The three regions with putative p63 binding sites and enhancer activity are highlighted. The predicted p63 binding motifs are shown below. **B** ChIP-qPCR analysis of p63 binding and H3K27ac enrichment at the enhancers from (**A**) in RT4 cells. A gene desert region was used as negative control for p63 and H3K27ac occupancy. Isotype control IgG were used as negative control for the ChIP. *n* = 3 (biological replicates). *P* by two-way ANOVA. **C** ChIP-qPCR analysis of p63 binding and H3K27ac enrichment at the enhancers from (**A**) in RT4 cells transfected with either scramble or pan-p63 siRNAs. Isotype control IgG were used as negative control for the ChIP. Values shown are fold enrichment over scramble for each region analysed. *n* = 3 (biological replicates). *P* by two-way ANOVA.

p63 is a well-established pioneer factor which recognises specific binding motifs and recruits epigenetic modellers to relax chromatin [30]. Since p63 was undetectable in muscle-invasive cell lines, we questioned whether epigenetic state of the *MIR205HG* locus was altered by the transition from the low stage to high stage tumour. We interrogated two publicly available H3K27ac ChIP seq experiments carried out in RT4 and T24 cell lines (Fig. 3A). Several H3K27ac-rich regions were also bound by p63 in various ChIP seq experiments from non-urothelial cells (Supplementary Fig. S3A). The *MIR205HG* locus had three regions enriched for H3K27ac mark and potentially bound by p63, highlighted as enhancer 1 (intronic +2.3 kb region), enhancer 2 (intergenic -13 kb region), and enhancer 3 (intergenic −17 kb region). Of note, p63 binding to the enhancer region 1 was previously described [27]. RT4 showed high levels of histone acetylation within all the three enhancer sites. It was reduced at enhancer 1 and partially enhancer 2 regions whereas enhancer 3 retained acetylation in T24 cells. Both enhancer 2 and enhancer 3 had canonical p63 binding motifs. We performed ChIP using anti-pan-p63, anti-H3K27ac or isotype control IgG and confirmed that p63 physically bound the three regions in RT4 cells (Fig. 3B). The loss of p63 by RNA interference led to a reduction of histone acetylation at enhancers 2 and 3 (Fig. 3C). Since the epigenetic state can affect recruitment of RNA Pol 2, we checked Pol2 occupancy at the enhancer sites and observed a dramatic decrease at all the three regions (Fig. 3C). Altogether, we demonstrate that p63 controls the epigenetic state of the *MIR205HG* locus.

### *LEADR* represses a subset of interferon stimulated genes

**Fig. 4:**
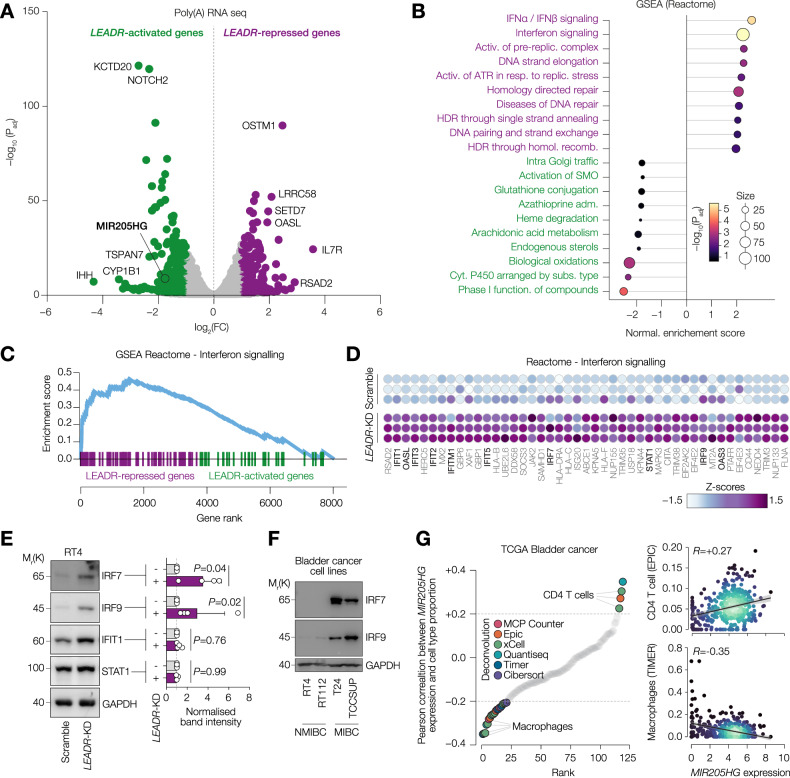
*LEADR* represses a subset of interferon stimulated genes. **A** Volcano plots showing mRNA differential expression in RT4 cells transfected with either scramble or *LEADR* siRNAs. Genes with abs(log_2_FC)>1 and *P* < 0.05 were considered significantly modulated. **B** GSEA Reactome pathway enrichment of modulated genes from (**A**). **C** GSEA Enrichment plot for the “Interferon signalling” pathway from (**B**). **D** Heatmap showing expression Z-scores of genes from the “Interferon signalling” pathway. **E** (Left) Western blot analysis of IRF7, IRF9, IFIT1, and STAT1 levels in RT4 cells transfected with either scramble or *LEADR* siRNAs. (Right) Band intensity analysis of the same samples. Values shown are fold enrichment over scramble normalised over GAPDH intensity for each protein. *n* = 4 (biological replicates). *P* by two-way ANOVA. GAPDH as loading control. **F** Western blot analysis of IRF7 and IRF9 levels in the panel of BLCA cell lines. GAPDH as loading control. **G** (Left) Dot plot showing Pearson correlation between *MIR205HG* expression and predicted proportion of infiltrating cells in TCGA BLCA samples based on publicly available deconvolution algorithms. (Right) Dot plots showing expression of *MIR205HG* and predicted proportion of either CD4 T cells or macrophages in TCGA BLCA.

To understand the role of *LEADR* in non-muscle invasive cancer, we performed knock-down of *LEADR* spliced transcript using a specific siRNA which does not affect *MIR205*-generating unspliced RNA as described before [29] and carried out poly(A) RNA seq. We identified 312 *LEADR*-activated and 235 *LEADR*-repressed genes (Fig. 4A and Supplementary Fig. S4A, B). Reactome gene set enrichment analysis (GSEA) revealed that *LEADR*-activated genes belonged to several metabolic pathways however with low level of confidence. On contrary, *LEADR*-repressed genes were strongly associated with interferon signalling and homology directed repair and replication stress (Fig. 4B). We first checked whether the loss of *LEADR* altered cell proliferation by performing growth curve analysis and EdU incorporation assay, yet we did not observe any significant changes (Supplementary Fig. S4C). We then questioned whether interferon pathway was affected by *LEADR* knock-down. Among interferon-related genes (Fig. 4C), we saw key transcription factors from STAT and IRF families and downstream targets, for instance, IFITs (Fig. 4D). We assessed protein expression of several hits and observed a significant increase in IRF7 and IRF9 protein levels in *LEADR*-depleted cells (Fig. 4E). Of note, IRF7 and IRF9 levels were dramatically increased in the two MIBC cell lines T24 and TCCSUP compared to non-invasive ones RT4 and RT112 (Fig. 4F). Since interferon pathway is a key signalling involved in immune response of tumours, we checked whether *MIR205HG* expression was correlated with any specific intratumoral cell types in bladder cancer by using distinct deconvolution methods. Tumours with high levels of *MIR205HG* were enriched for CD4 T cells and NK cells, on contrary *MIR205HG*^low^ tumours had a higher proportion of M2 macrophages (Fig. 4G). In summary, *LEADR* represses interferon-related genes and is associated with high CD4 T cells infiltration.

## Discussion

Here we identify a non-coding gene *MIR205HG* among the most enriched ncRNAs in non-muscle invasive bladder cancer. The primary transcript of *miR-205* host gene *MIR205HG* gives rise to a microRNA *miR-205* and a Long Epithelial Alu-interacting Differentiation-related RNA (*LEADR)*. We and others described the role of *miR-205* in various cancer types, including prostate [31–33], cervical [34], head and neck [35] cancer and melanoma [36], yet the role of the host gene remains understudied. Our data show that *MIR205HG* decreases during bladder cancer progression which is in line with a previous report [37, 38]. Indeed, *miR-205*, a product of *MIR205HG* gene, was shown to inhibit epithelial-mesenchymal transition through direct repression of ZEB1, ZEB2 and E-cadherin [38, 39].

Our screening of known transcription factors identified p63 as the most co-expressed with *MIR205HG* gene. p63, a member of the p53 transcription factor family, is essential for epithelial development and skin homeostasis [40–44]. In cancer, p63 exhibits context-dependent roles, acting as a tumor suppressor or oncogene depending on the isoform expressed [45–47]. In particular, the ΔNp63 isoform is crucial in bladder cancer, where it promotes epithelial identity and proliferation, and its dysregulation is linked to tumor progression and subtype specification.

Several reports showed p63 binding at the enhancer region (enhancer 1) within *MIR205HG* gene [29, 38] in prostate and bladder cancer. By assessing publicly available ChIP seq data and performing ChIP-qPCR assays, we showed that p63 bound additional −13 kb and −17 kb putative enhancers which harbour strong p63 motifs. p63 is a known pioneer factor able to recruit the chromatin remodellers to its binding sites to open chromatin. Contrary to a previous report [38], we did not see any changes of histone acetylation at the enhancer 1 after p63 knock-down, however, enhancer 2 and enhancer 3 H3K27ac levels were markedly reduced in absence of p63. Furthermore, all three regions lost RNA Pol2 binding in p63-depleted cells. It is plausible that the enhancer regions interact and form a loop allowing recruitment of Pol2 and active transcription of the gene. Since we observed p63 biding across the *MIR205HG* locus in the ChIP seq data from other tissues, it is likely that p63/*LEADR* regulatory axis is common for all epithelial tissues.

While the function of miR-205 has been thoroughly described, the role of the ncRNA transcript *LEADR* remains poorly understood. *LEADR* has been shown to affect cell proliferation in various cancers [48, 49] however we did not see any changes in cell growth or cell cycle upon *LEADR* knock-down in our model.

Of note, ZEB1/2, the miR205 targets in bladder cancer, were not affected by *LEADR* loss. Indeed, it was demonstrated that *LEADR* exerts a distinct miR-205-independent role in human prostate. By interacting with chromatin, *LEADR* binds to Alu sequences of *IL33* [50] and interferon stimulated genes (ISGs) [29, 51]. In this study, we performed an RNA sequencing of RT4 cells silenced for *LEADR* and observed a similar increase in expression of interferon α and β pathway genes. In fact, two key transcription factors IRF7 and IRF9 were significantly up-regulated also at protein level in *LEADR*-depleted cells.

Our analyses also indicate that the loss of *LEADR* may be associated with replicative stress and DNA damage response (DDR) which can stimulate activation of interferon pathway [52]. However, the enrichment of the DDR across *LEADR*-repressed genes was not strongly significant and we did not see any changes in cell proliferation. Therefore, it is plausible that *LEADR* directly represses interferon stimulated genes via the mechanism described previously [51]. Nonetheless, further investigation is needed to determine whether the loss of *LEADR* in bladder cancer cells can induce replicative stress and DNA damage, for instance, by analysing levels of phosphorylated histone H2AX and key proteins involved in DDR.

Interferon signalling is multifaceted, exhibiting both anti-tumour and pro-tumour activities [53]. A recent paper has shown that low grade bladder cancer cell lines are sensitive to IFNα treatment which induces expression of interferon stimulated genes and promotes tumour cell death [54]. On contrary, high grade cell lines are resistant to IFNα due to a constitutive activation of ISGs. This observation aligns with our findings, as we detected elevated levels of IRF7 and IRF9 in the muscle-invasive T24 and TCCSUP cell lines, but not in the non-muscle-invasive RT4 or RT112 cells. It is plausible that *LEADR* acts as an inhibitor of interferon response by preventing pro-tumoral chronic activation of ISGs [55]. Loss of *LEADR* in muscle-invasive tumours may be in part responsible for uncontrolled activation of the pathway and therefore development of resistance to immunotherapy. Since interferon response is a key signalling of the anti-tumoral immunity, we assessed the correlation between *MIR205HG* expression and immune infiltrate. In support of our hypothesis, deconvolution analyses revealed that tumours with high *LEADR* expression were enriched in CD4 T cells. Overall, our findings indicate that the non-coding RNA *LEADR* suppresses interferon signalling, thereby preserving tumour cell sensitivity to interferon and enhancing the anti-tumour immune response in bladder cancer.

## Materials and Methods

### Cell lines

Established human bladder cancer cell lines were purchased from American Type Culture Collection or Cytion. RT4 cells (ATCC; HTB-2) and T24 (ATCC; HTB-4) were cultured in McCoy′s medium (Gibco), RT112 cells (CLS; 300324) were cultured in RPMI medium (Gibco), TCCSUP (ATCC; HTB-5) cells were grown in Minimum essential medium (Eagle), and SW780 cells (ATCC CRL-2169) were cultured in Leibovitz’s L-15 Medium (Invitrogen). All the media were supplemented with heat-inactivated 10% Fetal Bovine Serum (Invitrogen) and penicillin/streptomycin (100 µg/mL, Gibco). Cells were maintained at 37 °C and 5% CO_2_ humidified atmosphere. Cells were routinely checked for possible mycoplasma contamination through MycoAlert Mycoplasma Detection Kit (Lonza).

### RNA interference

For transfection experiments, cells were seeded at density of 150.000 cells per well in a 6 well plate 24 h prior transfection. Then the culture medium was removed, and cells were transfected with specific siRNAs for 48 h, using Optimem medium (Thermo Fisher Scientific) and Lipofectamine-RNAiMAX reagent (Thermo Fisher Scientific) according to the manufacturer’s protocol. A control siRNA (scr, mission siRNA universal negative control #1 SIC001, Sigma) with no homology to any known human mRNA was used as a negative control. The siRNA used in the study are listed in the Supplementary Table S1.

### RNA isolation, reverse transcription and RT-qPCR

Total RNA was isolated from cells by using RNeasy Mini Kit (QIAGEN, Hilden, Germany) with DNase I digestion (QIAGEN, Hilden, Germany) according to the manufacturer’s protocol. RNA yield and A260/280 ratio were monitored with a NanoDrop 1000 spectrophotometer (Thermo Fisher Scientific Inc., Waltham, MA, USA). cDNA was synthesized using SensiFast cDNA Syntesis Kit with oligo-dT and random hexamer primers (Bioline). Real-time PCR was performed using the PowerUp SYBR Green Master Mix (AppliedBiosystems), in an QuantStudio™ 5 Real-Time PCR System (Applied Biosystems) with an amplification programme as follows: one cycle of 95 °C for 20 s and 40 cycles of 95 °C for 3 s and 60 °C for 20 s. GAPDH was used as housekeeping gene for data normalization. Relative expression was calculated by using the 2^−ΔΔCt^ method. The primers used in the study are listed in the Supplementary Table S1.

### Immunoblotting analysis

For total protein extraction, cells were lysed in RIPA lysis buffer (50 mM Tris-HCl pH 8.0, 150 mM NaCl, 1% NP40, 0.5% Na-deoxycholate, 0.1% SDS + protease and phosphatase inhibitors) for 10 min on ice and then centrifuged for 10 min to remove any debris. Proteins were denatured and negatively charged with Laemmli buffer and then boiled at 95 °C for 5 min. Proteins were loaded onto SDS-PAGE for separation and transferred onto polyvinylidendifluoride membranes. Membranes were blocked for 1 h in 5% milk and incubated overnight with shaking at +4 °C with primary antibodies: anti-p63 (Sant Cruz Cat# sc-25268, RRID:AB_628092, 1:500), anti-E-cadherin (Cell Signaling Cat#14472, RRID: AB2728770, 1:1000), anti-IRF7 (Cell Signaling Cat#13014, RRID:AB_2737060,1:1000), anti-IRF9 (Cell Signaling Cat#76684, RRID:AB_2799885,1:1000), anti-IFIT1 (Cell Signaling Cat#14769, RRID:AB_2783869,1:1000), anti-STAT1 (Cell Signaling Cat#14994, RRID:AB_2737027,1:1000), anti-GAPDH (Sigma-Aldrich Cat# G8795, RRID: AB1078991, 1:15000), and anti-β-actin (BioRad Cat#MCA5775GA, RRID:AB_2571580, 1:15000). After three washes with 1× PBS-Tween 20, membranes were incubated with the secondary antibodies conjugated to horseradish peroxidase (anti-mouse or anti-rabbit 1:10000) (Biorad Cat# 170-6515 RRID:AB_11125142 and Cat# 170-5047 RRID:AB_11125142) for 1 h at room temperature. Immunoreactivity was detected by immunodetection system (Alliance Q9 Advanced; Uvitec) with Luminol Reagent (Western Lightning Plus, Revity). The band intensity was measured using ImageJ software. The uncropped western blots are shown in the Supplementary Fig. S5.

### Immunofluorescence

Cells were grown in flat bottom 96 well PhenoPlates (Revvity) and then fixed in 10% formalin in 1X PBS for 10 min. Samples were washed twice for 5 min in 1X PBS. Cells were permeabilized in 0.25% Triton X-100 for 10 min. Five percent goat serum in PBS was used to block the samples for 1 h. Cells were incubated with primary antibodies diluted in 5% goat serum overnight at 4 °C. The wells were washed 3 times for 5 min in 1X PBS and DAPI (Sigma Cat# 11190301) and secondary antibodies were added to cells and incubated for 1 h. The plate was washed 3 times for 5 min in PBS. The samples were counterstained with 1 μg/mL DAPI (Sigma). The images were acquired on Operetta high content imaging system (Revvity) in confocal mode. The following antibodies were used: anti-p63 (Cell Signaling Cat# 39692S, RRID:AB_2799159, 1:200), anti-E-cadherin (Cell Signaling Cat#14472, RRID: AB2728770, 1:200), anti-mouse Alexa Fluor 647 (Invitrogen Cat #A-21235, RRID:AB_2535804), anti-rabbit Alexa Fluor 488 (Invitrogen Cat#A-11034, RRID:AB_2576217), phalloidin (Invitrogen Cat # A-12380).

### Chromatin immunoprecipitation (ChIP) analysis

To perform ChIP assay, cells were cross-linked with methanol-free 1% formaldehyde (ThermoFisher) for 10 min at room temperature. The formaldehyde was quenched by addition of freshly prepared 125 mM glycine (Sigma). After washing three times with PBS, cell pellet was resuspended in 0.5% SDS sonication Buffer (10 mM Tris pH8.0, 0.5% SDS, 2 mM EDTA) with protease inhibitor and incubated for 30 min on ice and fragmented (Covaris M220) by sonication. Size of the sheared chromatin was determined by agarose gel electrophoresis. Chromatin was diluted 1:10 in RIPA-LS buffer (Tris–HCl 10 mM pH8, NaCl 140 mM, EDTA 1 mM pH8, SDS 0.1%, Na Deoxycholate 0.1%) buffer. One percent of chromatin was preserved as input. The antibodies were added to the chromatin and placed in a shaker at 4 °C overnight. The following antibodies were used: anti-p63 (Cell Signalling Cat# 13109, RRID:AB_2637091), anti-H3K27ac (Abcam Cat# ab4729, RRID: AB_2118291), anti-Pol2 (ActiveMotif Cat# 39097, RRID:AB_2732926), mouse Gamma Globulins (31878) (Invitrogen Cat# 31878, RRID:AB_2532170). The day after, the Dynalbeads Protein G or Protein A (Invitrogen Cat# 10004D and 10001D) were added to the complex for immunoprecipitation for 2 h at 4 °C. The beads were washed twice in RIPA-LS buffer, twice in RIPA-HS buffer (Tris–Cl 10 mM pH8, EDTA 1 mM pH 8, Na-Cl 500 mM, Triton x-100 1%, SDS 0.1%, Na Deoxycholate 0.1%), twice in RIPA-LiCl buffer (Tris HCl 10 mM pH8, EDTA 1 mM, LiCl 250 mM, NP-40 0,5%, Na Deoxycholate 0.5%), and then in 10 mM Tris pH8.0. The immunoprecipitated chromatin was eluted in elution buffer (Tris-HCl pH8 10 mM, EDTA pH 8 5 mM, NaCl 300 mM, SDS 0.4%) with proteinase K for de-crosslinking and incubated at 55 °C for 1 h and then at 65 °C for 1 h. DNA was purified by QIAquick PCR purification kit (Qiagen), resuspended in nuclease free water and used for Real-Time PCR quantification (QuantStudio™ 5 Real-Time PCR System; Applied Biosystems). The genomic quantification was normalized to the input sample. The primers used in the study are listed in the Supplementary Table S1.

### RNA-FISH on cell lines

RNA-FISH was carried out as described in the protocol ViewRNA Tissue Fluorescence Assay (Invitrogen). Cells were fixed in 4% formaldehyde for 10 min and permeabilised in 70% ethanol for 1 h at 4 °C. RNA FISH probe was diluted in hybridization buffer 1:40, added to cells and then incubated for 2 h at 40 °C. Cells were washed twice for 10 min in Wash Buffer. RNA probes (diluted 1:25) were labelled with specific fluorophores (AlexaFluor-546 for *ACTB*; AlexaFluor-647 for *MIR205HG*) and incubated at 40 °C for 30 min. Finally, a counterstain was performed with 1 μg/mL DAPI (Sigma). Cells were washed two times with PBS and analysed on a high content imaging system Operetta (Revvity).

### Cell proliferation

Five thousand cells were seeded onto 96 well plates 48 h after knock-down and allowed to attach. The cell growth was assessed in an automated cell counter IncuCyte (Sartorius) using 10x objective and acquiring 5 fields per well every 4 h for 3 days. The proportion of the cells in S phase was estimated by fluorescence-based Click-it EdU incorporation assay (ThermoFisher) with AlexaFluor488 azide. The cells were incubated with 10 μM EdU for 1 h and then stained as per manufacturer’s protocol. The images were taken on the high content imaging system Operetta and quantified using Harmony software (Revvity).

### RNA-sequencing and analysis

Total RNA was isolated as described before. The amount and integrity of the extracted RNA were assessed by Agilent TapeStation instrument (Agilent Technologies, CA). RNA sequencing was performed in paired-end mode with a read length of 150nt using the Illumina NovaSeq 6000 (Illumina, CA) in paired-end mode (2 × 100 bp) with a coverage of 100 M reads. Reads were mapped onto a reference genome (human genome build hg19). The abundance of the transcripts was quantified using the Salmon algorithm. The differential gene expression was analysed with DESeq2 package [56]. The reproducibility of biological replicates was assessed by Principal component analysis (PCA). Differentially expressed genes are shown in Supplementary Table S2.

### Bioinformatic analyses

Cell line transcriptome data and TCGA data were downloaded from UCSC Xena Browser [57]and analysed using DESeq2 [56] and ggplot2 packages. The proportion of infiltrating cells were obtained from TIMER 2.0 [58]. Pathway enrichment was performed using fgsea package [59] and Reactome library. The list of known human TFs was downloaded from The Human Transcription Factors database. Volcano plots and correlation density plots were generated using ggplot2 package. Published ChIP seq data on H3K27ac were obtained from GSE213533 [60], while p63 ChIP seq data from ReMAP [61]. ChIP-seq signal tracks and peaks were visualised in UCSC Genome Browser. PCA plot was generated within DESeq2 package, while heatmaps were generated with pHeatmap package.

### Statistical analyses

All the statistical analyses were performed in GraphPad Prism 10. For the RT-qPCR and densitometry analyses, the significance of the differences between the groups were determined by the Student’s *t*-test or one-way ANOVA without adjustments. For the differential gene expression analysis, adjusted P values were calculated using DEseq2 package in R.

## Supplementary information

Supplementary Figures

Supplementary Table S1

Supplementary Table S2

## Data Availability

The RNA seq data have been deposited to NCBI GEO (accession: GSE293534).
