# Supplementary material for: LEADR, a p63 target, dampens interferon signalling in bladder cancer
Source: Cell Death Discov. 2025 Jun 3;11:264. doi: 10.1038/s41420-025-02546-1 (PMC12134291; doi:10.1038/s41420-025-02546-1)
Supplement: Supplementary file 1 — Supplementary Figures [file 41420_2025_2546_MOESM1_ESM.pdf]

Barnaba et al.

Supplementary Figures

Supplementary Figure 1

A

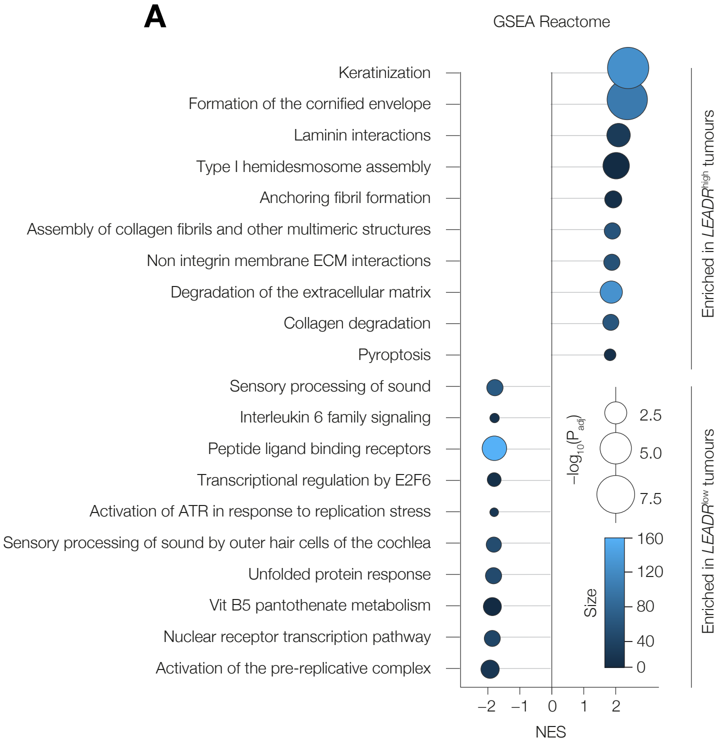

Supplementary Figure 2

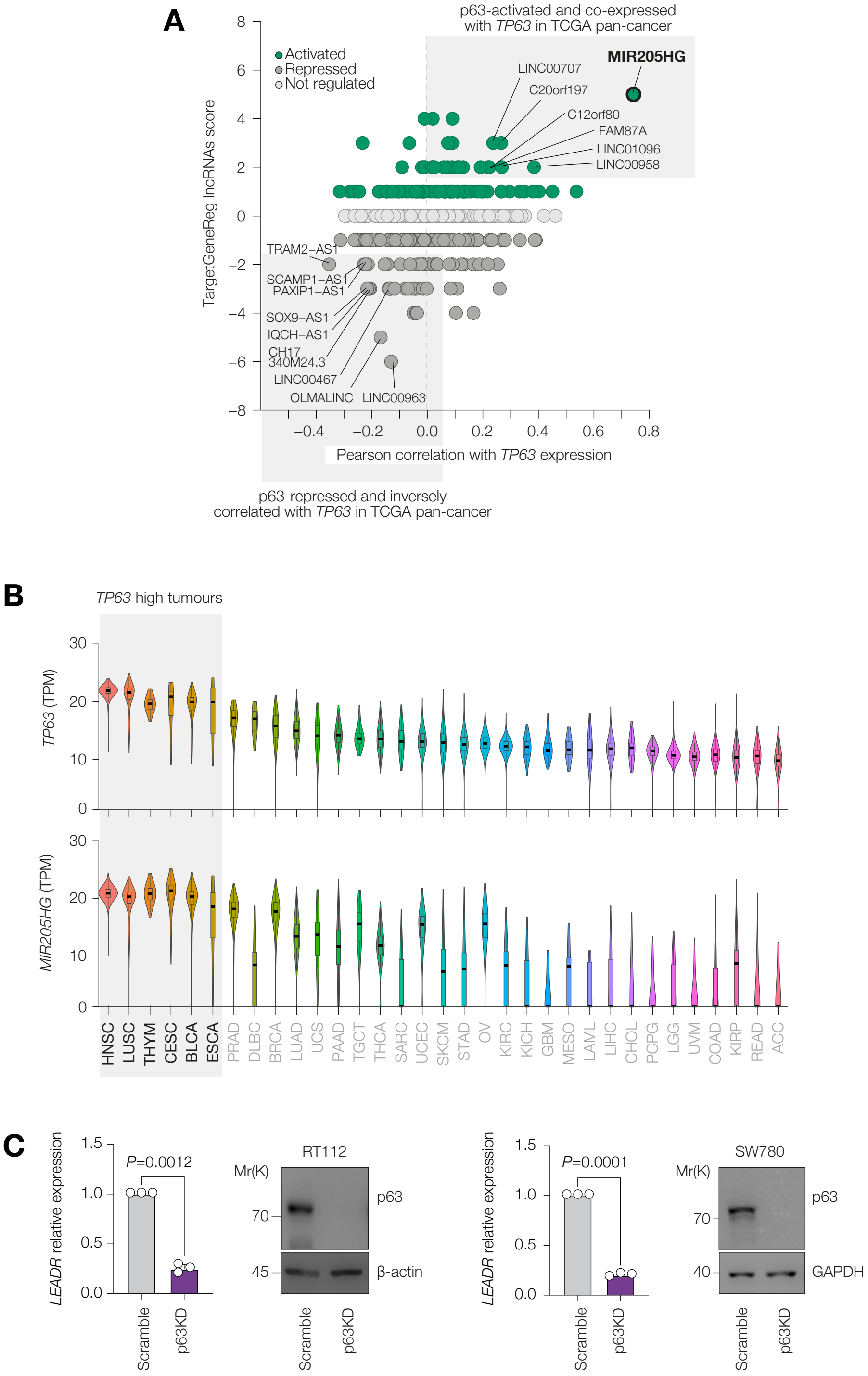

Supplementary Figure 3

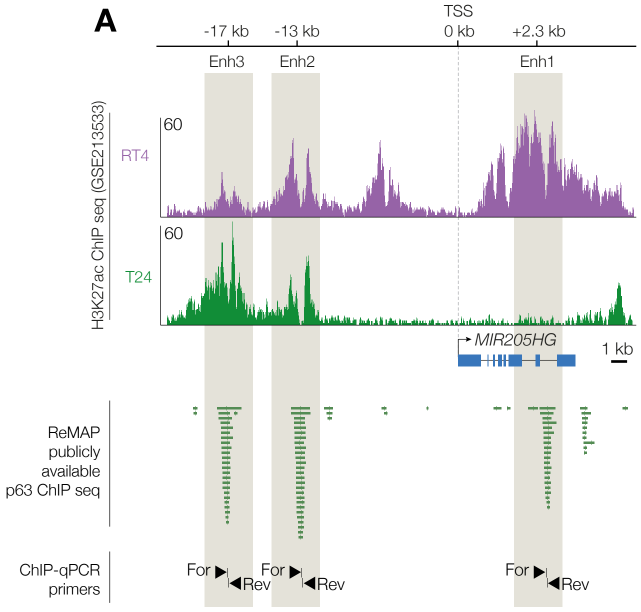

Supplementary Figure 4

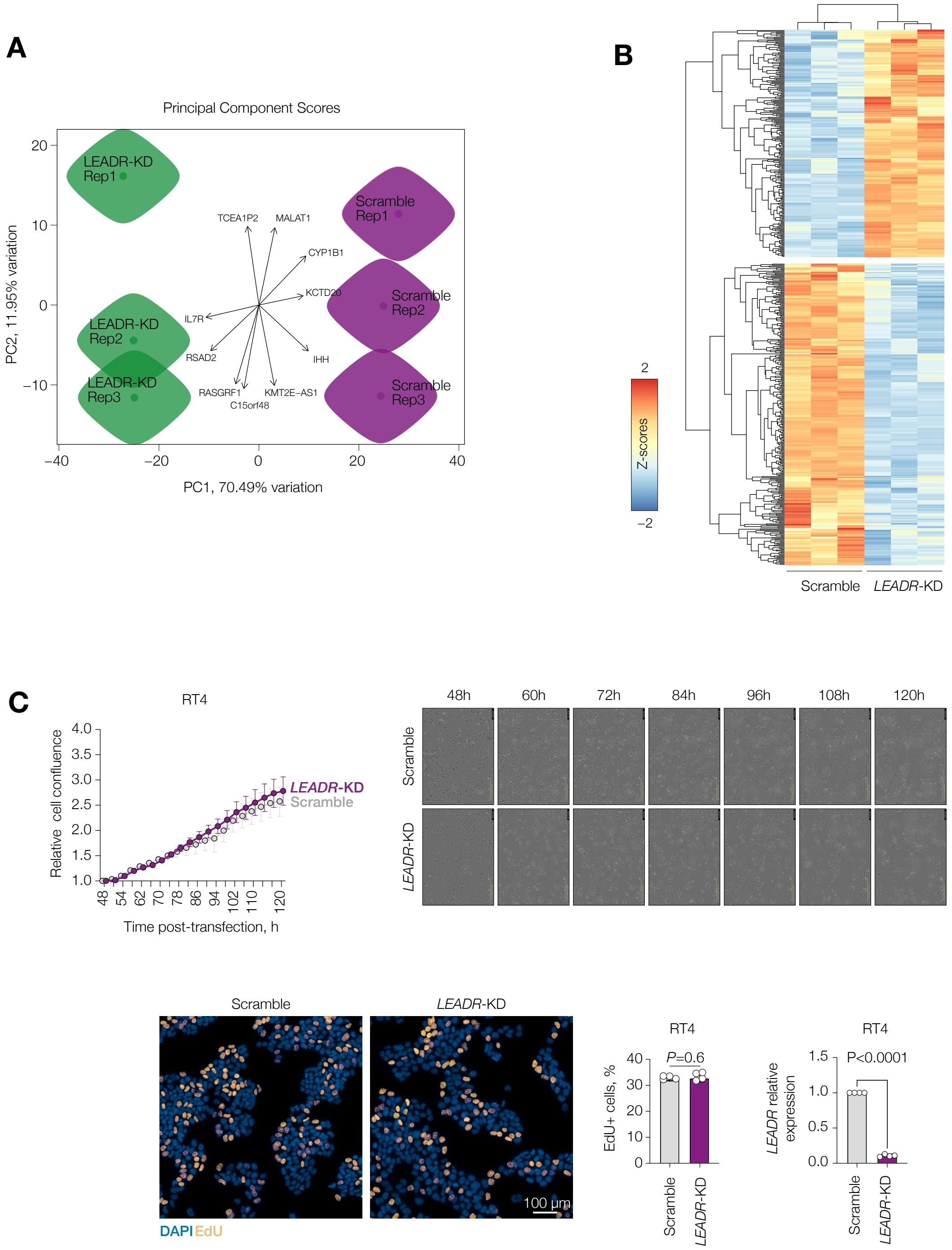

Supplementary Figure 5

Uncropped versions of western blots

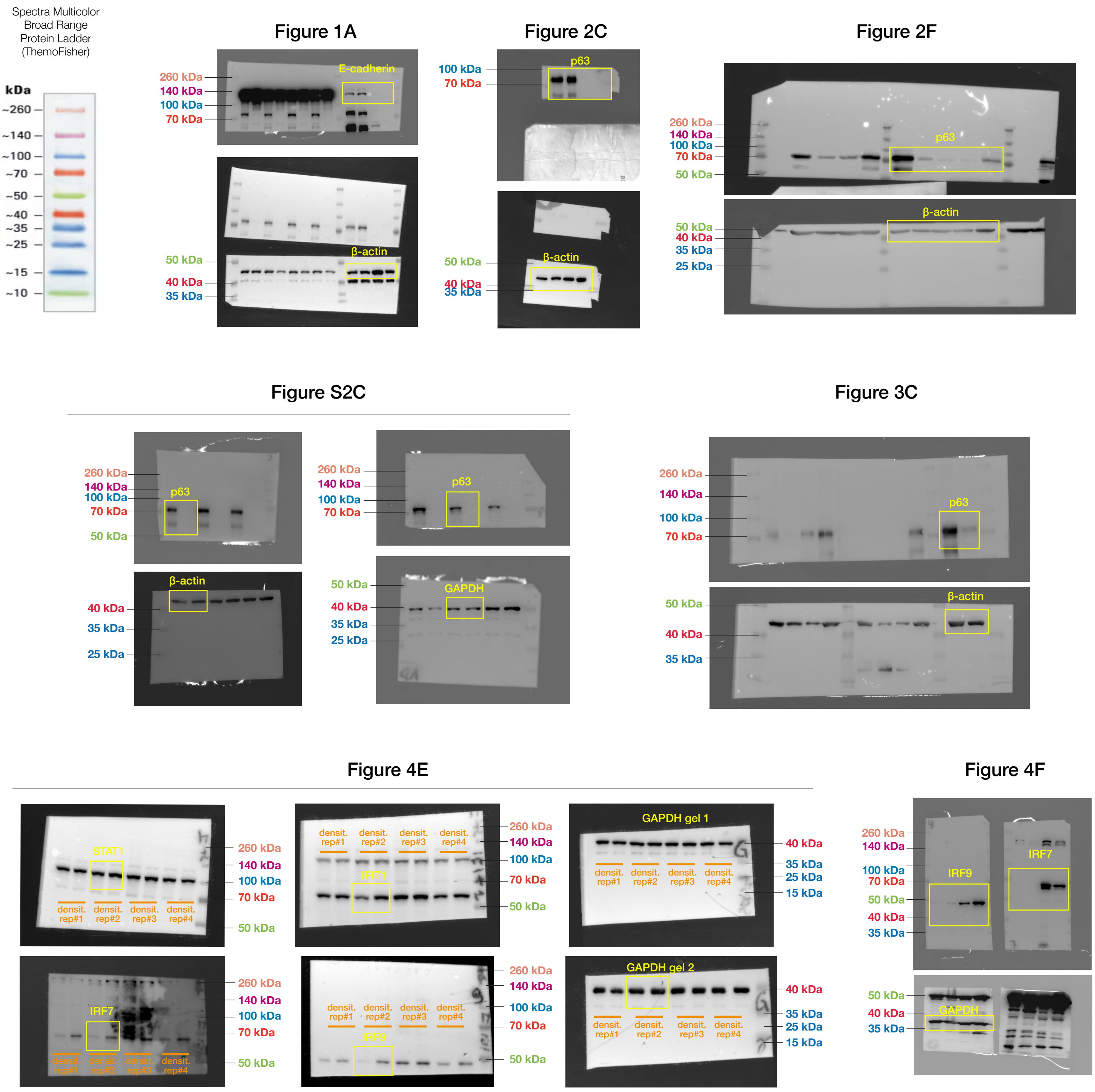

Supplementary Figure Legends

Supplementary Figure S1

(A) GSEA Reactome pathway enrichment of genes differentially expressed in *MIR205HG*-high vs *MIR205HG*-low TCGA BLCA tumours.

Supplementary Figure S2

(A) Dot plot showing distribution of ncRNAs based on their co-expression with *TP63* in TCGA Pan-cancer (Pearson correlation) and p63 target score from TargetGeneReg platform.
(B) Violin plots showing expression of *TP63* and *MIR205HG* in TCGA samples by cancer type.
(C) RT-qPCR analysis of *LEADR* expression in RT112 and SW780 cells knocked-down for p63 using distinct a pan-p63 siRNA. *n*=3 (biological replicates). *P* by unpaired Student's *t*-test. Western blot on the right confirms an efficient knock-down of p63. β-actin or GAPDH as loading control.

Supplementary Figure S3

(A) UCSC genome browser screenshot from Figure 3A showing p63 occupancy peaks identified in distinct publicly available ChIP seq experiments from ReMAP.

Supplementary Figure S4

(A) PCA plots showing distribution of samples used for the RNA seq.
(B) Heatmap showing Z-scores of the modulated genes in the RNA seq.
(C) (Top) Growth curves and (Bottom) % of EdU positive cells in RT4 cells transfected with either scramble or *LEADR* siRNAs. Representative brightfield or fluorescence images are shown. RT-qPCR on the right confirms an efficient knock-down. *n*=4 (biological replicates). *P* by Student's *t*-test.

Supplementary Figure S5

Uncropped western blots.
